# Racialised prescribing: enacting race/ethnicity in clinical practice guidelines and in accounts of clinical practice

**DOI:** 10.1111/1467-9566.12727

**Published:** 2018-04-06

**Authors:** Andrew Smart, Kate Weiner

**Affiliations:** ^1^ College of Liberal Arts Bath Spa University UK; ^2^ Department of Sociological Studies University of Sheffield UK

**Keywords:** ethnicity, race, drugs/medication

## Abstract

This article examines the articulation and enactment of racialised classifications in clinical practice guidelines and in accounts of clinical practice. It contributes to debates about racialisation in medicine and its consequences. The research centred on the case study of prescribing guidelines for hypertension in England and Wales, drawing on documentary sources and semi‐structured expert interviews. We found that conceptual and socio‐political uncertainties existed about how to interpret the designation ‘Black patients’ and about the practices for identifying patients’ race/ethnicity. To ‘close’ uncertainties, and thus produce the guidelines and treat patients, respondents drew authority from disparate elements of the ‘topologies of race’. This has implications for understanding processes of racialisation and for the future use of racialised clinical practice guidelines. We argue that clinical practice guidelines play a ‘nodal’ role in racialisation by forming an authoritative material connection that creates a path for translating racialised research into racialised healthcare practice, and that they carry with them implicit conceptual and socio‐political uncertainties that are liable to create inconsistencies in healthcare practice.

## Introduction

In certain contexts, clinicians in England and Wales are now guided to prescribe medicine based on a judgement about the patient's race/ethnicity.^1^ This practice raises sociological and normative questions, as racialised categorisations are socially sensitive and scientifically contentious. Current sociological interest in race/ethnicity and biomedicine is bringing together two overlapping bodies of scholarship. Work at the intersection of science and technology studies (STS) and the sociology of race/ethnicity has looked at the way racial/ethnic categories are instantiated in biomedical research, and in particular through their inclusion in genomic research. At the same time, sociologists of health and illness have a long‐standing engagement with race/ethnicity, including concerns about the causes and manifestations of ethnic inequalities in health, and the difficulties that can arise from intervening in these. Through our research on the development and practices of ‘racialised prescribing’, we contribute to this scholarship, and specifically to debates about the risks and benefits of using racialised classifications in medicine and to sociological knowledge about the role of health care in articulating and enacting racialised categories.

While there is a growing body of biomedical research that differentiates patients and/or participants racially, and an accompanying growth in social science examinations of these, there has been little examination of whether or how these matters are translating into clinical practice. In this article we examine a case study of clinical practice guidelines (CPG) for hypertension in England and Wales that recommend racialised distinctions in prescribing. We report on our analysis of documents (including guidelines, the studies cited in those guidelines and stakeholder consultations) and semi‐structured interviews with experts. This allows us to address questions about the development and usage of a CPG that asks clinicians to make racial/ethnic differentiations in their practice. To analyse our data we apply, for the first time in a clinical context, a ‘topological account of race’ (M'charek 2014: 48). This helps us to explain how conceptual and socio‐political uncertainties are ‘closed’ to allow the guideline to be produced and implemented, and allows us to develop ideas about racialisation processes.

## Background

Against a backdrop of policies that require health service providers to recognise and address health inequalities, the UK has long‐standing racial/ethnic variations in health (Nazroo 2010). In the context of our research, there is reasonably consistent evidence of higher blood pressure and an increased prevalence of hypertension among people from a range of minority ethnic groups (Agyemang and Bhophal 2003, Lane and Lip 2001). Those in the Black African and Black Caribbean ethnic groups, and women who identify as Bangladeshi, have been shown to have the most elevated risk (Sproston and Mindell 2006). Variations in health outcomes as a result of hypertension are also linked to race/ethnicity, including increased likelihoods of stroke in Black British patients, heart disease in British Asian patients and chronic kidney disease in both Black British and British Asian patients (Brown 2006).

Sociologists of health have long engaged with conceptual questions about race/ethnicity, alongside examining patterns of inequalities, racism, patient experience, and frameworks for policy and practice (Ahmad and Bradby 2007, Nazroo 1998). Debates about whether race/ethnicity should be conceptualised in biological, social or cultural terms (or as some hybrid of these) gain a hard, practical edge when the answers have implication for clinical practice (Braun *et al*. 2007), biomedical research priorities (e.g. Montoya 2011) or how to measure and address health inequalities (Aspinall 2001, Bradby 1995, 2003). One underlying issue is that the benefits of addressing racial/ethnic inequalities are attended by a risk of reifying ideas about fixed, homogenous and ‘othered’ groups. This raises fears about medical errors due to misjudgements about race/ethnicity, and concern that differentiated health interventions will contribute to essentialised ideas about group difference (Braun *et al*. 2007, Duster 2015). The paradox of race as simultaneously oppressive and emancipatory has deep historical roots (Schramm 2015). It finds contemporary expression in the biopolitics of ‘inclusion and difference’ (Epstein 2008), where a conjunction of the state's responsibility to deliver healthcare equality with scientific endeavours to understand population‐level variation has aligned socio‐political classifications with biomedical research and created particular problematisations of populations at risk. It has been argued that there is a tendency to naturalise socio‐political groups and, in an era dominated by genomic science, to biologise (or geneticise) the causes of health differences between those groups (e.g. Duster 2015).

One preoccupation of those examining how genomics intersects with race/ethnicity has been differences between contemporary and historical racialised science; asking whether we are witnessing an entrenchment of old ideas (e.g. Duster 2015, Skinner 2007). One contemporary inflection, revealed by empirical studies, is the interweaving of equality discourses into some genomic research (e.g. Fullwiley 2008, Montoya 2011); Bliss (2012), for example, argues that an ‘anti‐racist racialism’ is advanced when scientists use racial categories to unsettle biological narratives of race or to address ethnic health inequalities. However, such studies also demonstrate how racialised thinking shapes genomic knowledge production, and the authors express unease about the geneticisation of racial/ethnic categories and the social consequences of this. These ambiguities are not unique to genomics: similar debates accompany the ‘ritual’ use of race/ethnicity as a variable in epidemiological research (Shim 2014), and they framed discussion when a heart failure drug – BiDil – was licensed for ‘self‐identified Black patients’ in the US (Kahn 2012). This case sparked acrimonious debate about balancing concerns about the reification of race/ethnicity with contemporary opportunities for addressing health inequalities (Pollock 2012).

Research about how this emerging science translates into clinical practice is sparse, but it appears that practitioners also face conceptual and socio‐political uncertainties. Bonham *et al*. (2009) found US clinicians were wary of making simplistic connections between genes, race/ethnicity and health; while in contrast, Hunt *et al*. (2013: 267) reported clinicians often ‘painted minority patients with a broad brush, drawing on a variety of presumptions’. Both Bonham and colleagues and Hunt and colleagues found that US clinicians thought that race/ethnicity is relevant in the clinic, but there was a lack of agreement about what this meant for practice. In England, Dyson *et al*. (2007) document how the assumptions and understandings of health practitioners and clients influenced constructions of ethnicity in a genetic screening programme for sickle cell/thalassaemia. For example, some midwives appeared to have a belief in distinct racial groups and others over‐rode a standardised procedure for coding ethnicity by assigning patient ethnicity based on visual cues like skin colour. We contribute to the above body of scholarship by linking racialised research, CPGs and clinical practices, using the case of hypertension.

Pollock (2012) shows that high blood pressure is a racialised disease category in the US, and furthermore, she argues that being able to take action in the clinic involves ‘closing’ intractable problems about the nature and role of race. She argues that ‘African American Hypertension’ is characterised by ‘epistemological eclecticism’ (Pollock 2012: 2) that reflects the variety of aetiological factors for which race stands as a proxy (including variations linked to socioeconomic status, poverty, diet, lifestyle, culture, stress, racism, biology or genetics). Pollock explains that it is even less tenable for physicians than epidemiologists to separate these factors. Thus, a common rhetorical move ‘in the field of race and medicine’ involves opening up the difficult conceptual and/or socio‐political questions about race, only to then ‘close the conversation to allow implementation of operational answers’ (Pollock 2012: 84). This opening and ‘closing to operationalise’ reflects the fact that while clinical medicine is concerned to understand the causes and distribution of disease it also ‘strives to determine the course of action to be pursued at the patient's bedside’ (Pollock 2012:84). The process of closing is a necessary step that facilitates action in the face of a perceived problem.

Given these contexts, biomedical science has become a key site for examining racialisation to better understand its processes, dynamics and consequences (M'charek *et al*. 2014b, Duster 2015). Attending to racialisation involves analysing the social processes through which ideas of racial/ethnic difference gain meaning or salience (e.g. Omi and Winnant 1994). We adopt a ‘topological approach’ that enables us to focus on questions about how racialised populations are materialised (M'charek *et al*. 2014b). M'charek (2014) argues that race is enacted in objects and practices rather than being fixed in biology or ideology. From this perspective, ‘race gets assembled in specific historical contexts by associating different kinds of entities’ (M'charek *et al*. 2014a: 461). Assembling or enacting race involves folding together elements – ideas, practices, artefacts and social relations – that can be spatially and/or temporally distant (M'charek 2014, M'charek *et al*. 2014a, 2014b, Schramm 2015). Consequently, race ‘moves and changes shape depending on the times and places that are drawn together’ (M'charek 2014: 48). Using this approach it becomes possible to examine how race is ‘made up’ in specific contexts, and to map ‘the epistemic genealogies and multiple references’ that enable it to be meaningfully assembled (Schramm 2015: 53). We show the elements of race that are enfolded in the creation and use of the CPG for hypertension.

To summarise so far, while ethnic health inequalities are well documented, the relationship between race/ethnicity and health is rife with uncertainties and differentiated health care carries a risk of reification. Biomedicine is thus an important and useful site for considering racialisation and its consequences. Despite much recent work on constructions of race/ethnicity in genomic research, relatively little attention has fallen on clinical practice. To address this gap we focus on a CPG for hypertension in England and Wales, which, as we explain in the following section, has introduced racial/ethnic differentiation in prescribing pathways. When we discuss our data, we reflect on how uncertainties about race/ethnicity in the guidelines were ‘closed’ to enable action, and how the disparate elements that get assembled in and around the guidelines lent authority to these closures. We show that our novel adoption of a ‘topological account of race’ (M'charek 2014: 48) can help to explain the articulations of racial/ethnic difference that occur in a clinical context, and use this analysis to suggest that authoritative artefacts like CPGs play a ‘nodal’ role in racialisation processes.

## Methods

Our case study examines the development and implementation of guidelines for the treatment of hypertension. In 2006, the National Institute for Health and Care Excellence (NICE), the key provider of CPGs in England and Wales, updated its guideline on hypertension, introducing a prescribing algorithm differentiated by age and race/ethnicity (NICE 2006). This approach was maintained in slightly amended form in the revised 2011 guideline (NICE 2011). The prescribing algorithm introduced in 2006 recommended that, as a first line treatment, people ‘55 years or older or Black patients of any age’ should be prescribed either a calcium channel blocker or a diuretic, whereas, ‘people younger than 55 years’ should be prescribed an ACE inhibitor or an angiotensin receptor blocker (ARB) (NICE 2006: 45). Health professionals are ‘actively encouraged’ to follow such clinical guidelines, although these are recommendations not obligations, and are not intended to replace ‘clinical expertise and professional judgement’ (NICE 2017). In line with NICE's standard process, an expert Guideline Development Group developed the hypertension guidelines. Such groups draw on commissioned reviews of evidence to produce evidence‐based recommendations for practice. In the NICE (2006) hypertension guideline, the prescribing algorithm was derived from clinical trials evidence that was interpreted to show that different drugs could be more effective in different sub‐populations, and from evidence from other types of studies that was used to explain these differences in effectiveness by combining ideas about drug mechanisms and racially/ethnically differentiated disease pathologies.

The wider aims of our research were to understand: what ideas about race/ethnicity underpinned the guidelines and what research informed them; what discussions or controversies, if any, were associated with the development of the guidelines; and what experts thought about the implementation of the guidelines in practice and any difficulties that might arise.

The research involved documentary analysis and expert interviews. Documents included: (i) three iterations of the NICE hypertension guidelines (North of England Hypertension Guideline Development Group 2004, NICE 2006, 2011) and associated materials concerning their development and implementation available on the NICE website; (ii) biomedical and trials literature cited as evidence for the racial/ethnic differentiation within the guidelines (23 articles); (iii) responses to the NICE stakeholder consultation on the draft guidance (NICE n.d.); and (iv) discussions about the guidelines published in medical journals, in particular the rapid responses to papers in the BMJ that accompanied the launch of the 2006 and 2011 guidelines. We reviewed these documents paying particular attention to the ways race/ethnicity were defined and discussed and awareness or discussion of the sensitivities and uncertainties relating to race/ethnicity.

We also undertook 11 semi‐structured expert interviews. Our aim was to elaborate on our understandings of the guideline development and any attendant discussions and controversies that we had identified from the published literature. The interviews provide an opportunity to supplement our documentary analysis with any tacit or unreported facets. Interviews were also used to explore the nature of concerns that might exist about the implementation of the guidelines and how these might be resolved.

Participants were purposively selected to include a range of expert viewpoints. We approached 24 potential interviewees, identified through membership of the Guideline Development Groups, through having commented on the guidelines or published on race/ethnicity and hypertension in the UK context in clinical journals, and through snowball sampling. We were careful to include experts from a range of disciplinary backgrounds, including clinical expertise in cardiovascular disease, primary care, clinical pharmacology, public health and epidemiology. Nine interviewees were clinicians, three of whom had been members of a Guideline Development Group. We do not provide more specific details on the exact recruitment and identity of different interviewees for the sake of anonymity. The research gained ethical approval through institutional review at Bath Spa University.

Interviews were undertaken face‐to‐face (3) or by telephone (8) and lasted for 35–75 minutes. All were digitally recorded and transcribed. Interviews were semi‐structured, guided by an interview prompt that covered the same broad topics. They were tailored to the specific contribution of different participants to either guideline development or public discussion of this, and to their areas of expertise (including as clinicians, or not). In each case it covered participants’ ideas about the basis for the differentiated prescribing pathway; whether they saw any controversies or difficulties with the evidence or guidelines; how guidelines were, or should be, applied in practice, including questions about how to judge patients’ race/ethnicity; and whether they related this to wider questions about equality in health care. We recognise that interview data do not provide a straightforward ‘window’ onto the enactment of the CPGs in practice, but they do reveal these experts’ accounts of their practices, or for the non‐clinicians, their opinions about clinical practices.

Interview data was analysed following a ‘framework’ approach (Ritchie and Spencer 1994), whereby both authors reviewed transcripts to identify key themes, which were then applied cross‐sectionally to compare differences between respondents. In the analysis presented in this article we concentrate on the different articulations and enactments of race/ethnicity that emerge across the data as we moved from evidence to written guidance through consultation to expert accounts of how these would or should be implemented in practice.

## Findings

Our analysis focuses on: (i) the articulation of race/ethnicity in the CPGs and the discussions surrounding these; and (ii) the enactments of race/ethnicity in interviewees’ reflections on and accounts of clinical practice. We highlight for later discussion the disparate elements of the topologies of race that get assembled to articulate and enact race/ethnicity, and the sources of authority that are invoked to ‘close’ the conceptual and socio‐political uncertainties that emerge.

### Articulation in the guideline: a science‐state hybrid

A systematic search for scientific evidence of treatment efficacy and a formalised stakeholder consultation are central to the process for developing NICE guidelines (McManus *et al*. 2012). When the draft of NICE guideline CG34 was circulated for consultation, it recommended a differentiated prescribing pathway for ‘patients aged 55 or over, or Black patients of any age’. We show: (i) that this recommendation was underpinned by clinical trials evidence from the US; (ii) that when some stakeholders struggled to interpret the designation ‘Black patients’ used in that evidence, NICE initially closed uncertainty by invoking a bureaucratic requirement to use an ethnic taxonomy from the census of England and Wales; (iii) that the articulation of race/ethnicity in the final guideline folds together these elements into a hybrid designation of a racialised ‘target’ for prescribing practice.

To understand these articulations of race/ethnicity, it is useful to reflect on the origins of the designation ‘Black patients’ in the draft guideline and how this relates to an apparently decisive set of scientific evidence. While a racialised science of hypertension reaches back to the 1930s (Jamerson and DeQuattro 1996), the NICE (2006) guideline cited the 1994–2002 Antihypertensive and Lipid‐Lowering Treatment to Prevent Heart Attack Trial (ALLHAT) to show evidence of racial differences in clinical outcomes. ALLHAT was a US clinical trial that compared the effectiveness of different antihypertensive drug regimens (The ALLHAT Officers and Coordinators for the ALLHAT Collaborative Research Group 2002). In an effort to address questions about race‐based health inequalities, this state‐funded study recruited large numbers of participants from minority ethnic groups. In ALLHAT's protocol, a participant's race was defined by self‐report as Black, White, Asian, native American, and other (Wright *et al*. 2005). In key publications, however, the last 4 categories are combined so that findings are presented as a comparison of Black patients versus non‐Black patients (Wright *et al*. 2005). As NICE guideline CG34 cites evidence from ALLHAT to support its racialised approach, the designation ‘Black patients’ appears linked to these citations.

A number of aspects of the topologies of race can be illustrated here. ALLHAT's deliberate over‐sampling of participants from minority ethnic groups illustrates that socio‐political concerns have been folded into the practice and outcomes of science, exemplifying Epstein's (2008) ‘inclusion and difference’ paradigm. The use of US clinical trials evidence in the NICE guidelines exemplifies how elements, such as the designation ‘Black patients’, are drawn from place to place (from the US to England and Wales), and from context to context (research studies to clinical practice).

When the draft guideline was circulated in a consultation exercise, however, a wide range of stakeholder organisations made comments about its use of race/ethnicity, including national clinical professional organisations (Royal College of General Practitioners (RCGP), Royal College of Nursing (RCN), Faculty of Public Health), health charities/ interest groups (Blood Pressure Association (BPA), Diabetes UK, South Asian Health Foundation), academic experts (University of Birmingham, Department of Primary Care & General Practice), industry (Merck Sharp & Dohme Ltd) and healthcare providers (North Sheffield Primary Care Trust (PCT), Rotherham PCT). The most frequently expressed issue (seven instances) related to the clarity, definition or meaning of the designation ‘Black patients’. For example, the BPA asked: ‘who falls into the category of Black’ and the RCN questioned: ‘is it restricted to patients who are African, Afro Caribbean, or to patients who are of mixed race origin?’ (NICE n.d.). These question show that stakeholders’ were grappling with the implication that ‘Black patients’ was a concept that would need to be interpreted and operationalised in practice, although it is also notable that these comments are not directly about the clinical validity of racialised prescribing per se.

This focus on practicality was also accompanied by expressions of broader socio‐political and ethical concerns, including questions about how to treat other minority ethnic groups. Six out of the seven questions about ‘who counts as Black’ were accompanied by a question about how to treat ‘South Asians’. The term ‘South Asian’ is used in the UK, not least in health research, as a pan‐ethnic category encompassing those with Bangladeshi, Indian or Pakistani origins (among others). Indeed, one of the stakeholder organisations that raised this question exists to promote ‘good health in the UK's South Asian communities’ (http://www.sahf.ord.uk). Concerns about clarity for clinical practice thus overlap with issues of equality, inclusion and representation. As such, some stakeholders argued that specific minority ethnic groups should be named in the guideline, even, as the RCGP suggested, when ‘such mention may merely be to state that evidence is lacking’. Another socio‐political concern (raised twice) was sensitivity to terminology; as the BPA noted: ‘The word Black may also be viewed by some as an offensive or insensitive term’ (NICE n.d.).

Our expert interviews also provided evidence of similar socio‐political sensitivities. For example, two respondents thought that racial/ethnic difference was a sensitive or ‘taboo’ (Respondent 10) topic for research, and that this contributed to a lack of funding. Four interviewees advocated ideas that promoted racial/ethnic equality, although there were differences in their viewpoints about whether this meant paying more or less attention to race/ethnicity. One feared that by ‘categorising people along [racial/ethnic] lines you create social division and that probably will also lead to discriminatory, you know discrimination’ (Respondent 4); but another argued against being worried about the use of race/ethnicity because: ‘you can build in institutionalised or structural racism by reifying *out* diversity and saying everybody is the same’ (Respondent 5).

In its documented reply to the stakeholder consultation, NICE accepted responsibility for clarifying its terminology. Collating the various comments under a ‘combined response to duplicate questions’, its summary of the key questions and its response to these read:

Should the guideline explain which patients are Black and which are not? Should the term be used at all? It is NICE policy to use the ethnic taxonomy used by National Statistics in the census. However, as various stakeholders have expressed concern about how to interpret this, the developers have added a footnote to 1.5.1.1: “Including both Black African and Black Caribbean patients, but not Asian, Chinese or other ethnic groups”. (NICE n.d.: 126)

Stakeholders’ conceptual/operational and socio‐political concerns were thus addressed by deferring to a policy of using an ethnic taxonomy from state bureaucracy and by specifying certain categories. We do not know whether this response addressed the concerns of stakeholders, but it does raise other awkward questions about clinical validity. For example, is it legitimate to treat those who self‐identify to the census categories ‘Black African’ and ‘Black Caribbean’ in England and Wales as if they were the same as the ‘Black patients’ in the US clinical trial?

The published guideline did not, in the end, actually use the above‐noted census categories (Black African and Black Caribbean) but instead created a ‘hybrid’ phrasing that specified the population to be targeted: ‘Black patients are those of African or Caribbean descent, but not mixed‐race, Asian or Chinese patients’ (NICE 2006: 45). This is a notable departure from the NICE policy stated in the stakeholder response document that raises two issues that we discuss further in the next section. First it added ‘mixed‐race’ as a specifically excluded patient population, and second, it introduced the notion of ‘descent’.

When we asked those who had been involved in guideline development whether race/ethnicity was controversial in this process, they recalled issues of terminology but did not consider these a significant concern. When prompted to comment on how these issues were resolved, one respondent appeared exasperated by the wrangling over language. He remembered ‘almost losing the will to live’ when it was discussed, as ‘everybody knew what we were talking about’ (Respondent 1). Another respondent appeared more disengaged and claimed a professional delineation: ‘the committee had made the decision and how that actually got represented was something that could be hammered out by the draughtsmen’ (Respondent 2). However, as he then went on to explain: ‘NICE wanted to be sure that the wording matched the evidence and was politically correct’. We interpret the usage of the term ‘politically correct’ in this context to be somewhat disparaging, signaling that group labels were a politicised and bureaucratically imposed concern that was separable from the scientific evidence. For these Guideline Development Group members, the issue of racial/ethnic nomenclature appears to have been both uninteresting and a source of frustration or annoyance. Their responses suggest perceptions of an overbearing socio‐political or bureaucratic concern with terminology, which had to be appeased. This discordance arises, we suggest, because the actors involved invoke their authority for ‘closing’ the uncertainties of race/ethnicity from different realms: from science or from state bureaucracy.

It is notable, however, that folded into scientific and bureaucratic standpoints, and more broadly in stakeholder responses, were ‘common‐sense’ notions of racial/ethnic difference: difference that is deemed manifest, obvious and uncontroversial (Posel 2001a, 2001b). The draft guideline had labelled ‘Black patients’ as if this category was self‐evident, an assumed consonance that was encapsulated in the remark: ‘everybody knew what we were talking about’. Furthermore, state classifications ‘perpetuate “conventions” of race already ingrained in the social fabric’ (Posel 2001a: 104). In our case, the census categories adopted by NICE are themselves contingent on British colonial history and are not part of the everyday or bureaucratic lexicon of racial/ethnic difference elsewhere in the world. Similarly, while the designation ‘South Asian’ is not a census category, it was part of the locally meaningful working vocabulary of UK health organisations, which included an interest group that represents the needs of that named population.

The articulation of race/ethnicity in the final guideline can be understood as a folding together of such disparate elements from the topologies of race. Designating a racialised ‘target’ population in the guideline brought into proximity evidence from a US clinical trial and a bureaucratic census tool for England and Wales. This happened via a guideline development process in which stakeholders raised conceptual/operational and socio‐political questions (but not, overtly at least, questions about clinical validity). The resulting articulation of race/ethnicity in the guideline is a hybrid that folds together elements of race/ethnicity that originate from various times and places. However, the discordance noted by respondents involved in guideline development highlights that claims to speak authoritatively about race/ethnicity were being negotiated, ostensibly between those representing science and those representing the state. Nonetheless, these authoritative standpoints and their negotiations are themselves informed by and interlaced with ‘common‐sense’ ideas about racial/ethnic difference.

### Classification in practice: self‐identity, phenotype and population prototypes

In our interviews we further explored the existence and nature of concerns about applying the CPGs in practice (non‐clinicians were asked to offer their view on potential concerns). In presenting our findings we draw a contrast between how interviewees answered questions that were framed openly and their responses to questions we posed about liminal cases that were designed to unsettle or breach the classification scheme in the guideline.

We asked respondents how they identified a patient's race/ethnicity in practice or, if they were not clinicians, how they would advise clinicians to do so. They suggested two main methods: (i) asking patients to self‐identify their race/ethnicity; and (ii) visual identifications, an approach described as ‘eyeballing’ (Respondent 5) or ‘how people look’ (Respondent 9). None of the respondents suggested or implied that either identification process was considered problematic in practice, although socio‐political sensitivities were evident. Some respondents voiced a tacit understanding that visually identifying a patient's race/ethnicity might not be considered a socially or professionally appropriate strategy. For example, a non‐clinician distinguished between what he thought should ideally happen (asking) and what he thought often does happen (visual identification). One clinician commented that it might be ‘a crude thing to say, but most of the time I guess it's quite clear’ (Respondent 7). While this could mean that a visual classification was rudimentary (i.e. that it is crude to claim that people can be identified racially/ethnically based on their appearance), the expression that it was ‘a crude thing *to say*’ implies that visually identifying race/ethnicity is in itself a coarse or vulgar activity. Another clinician acknowledged the potential sensitivities, but argued that these were alleviated by the context: ‘I suppose there's always that political correctness thing isn't it, are you afraid to insult anybody, but I think in the sort of healthcare setting I think people are less concerned about it because you have to get to the answer before you can treat people’ (Respondent 9).

In these data we can see different elements of the topologies of race being used to close uncertainties about a patient's race/ethnicity. There is a standardised technique drawn from the national census, whereby respondents are requested to self‐identify from a classification scheme that (ostensibly) marks socio‐political identities. By using this practice, health professionals invoke the authority of state bureaucracy. Another element is the practice of observer‐assigning race/ethnicity based on phenotypical differentiations like skin colour, hair texture, etc.. We argue that these visual judgments, about ‘how people look’, close uncertainties by invoking authority from ‘common‐sense’. This is not only because they adopt ideas about racial/ethnic difference that are based on ‘social notions of phenotypical difference’ (M'charek *et al*. 2014b: 13) but also because some respondents considered such judgments to be ‘obvious’ (Posel 2001a, 2001b).

To test the idea that, as one respondent expressed it, ‘most of the time’ racial/ethnic identifications were ‘quite clear’, we asked interviewees to reflect on instances that might challenge the boundaries of the category ‘Black patients’. In particular, how might ‘mixed‐race’ and North African patients be identified and treated? The issues pertaining to ‘mixed‐race’ and North African patients were rarely brought up by respondents, but once raised were readily acknowledged as ‘difficult’, or ‘interesting’ or ‘not straightforward’. We show that respondents closed uncertainties about such cases by recourse to ideas about ‘pure’ populations – or ‘population prototypes’ – that draw authority from both ‘common‐sense’ and science.

Once the issue of ‘mixed‐race’ was raised, some respondents warmed to it, and volunteered examples of patients who might not easily fit the guideline classification. When asked about applying the guidelines, two clinicians said they would treat ‘mixed‐race’ patients as if they were ‘Afro Caribbean’, thereby categorising them as ‘Black patients’. Such a decision actually runs contrary to the prescribing recommendations of the NICE (2006) guideline. Underlining her sense that this question called for an active judgment, one of these respondents said: ‘I think probably I'd have to nail my colours to the mast one way or another and I would probably classify them as being Afro Caribbean for the purposes of prescribing’. However, she also implied a stance of critical flexibility by adding the caveat: ‘and then I would monitor the effect of the treatment and see how they got on with it’ (Respondent 7).

These respondents address uncertainties about ‘mixed‐race’ by deciding which ‘component’ in a combination should be prioritised. Interpreted topologically, this practice draws on ideas about race and mixture that invoke historical populations of ‘origin’ (Fullwiley 2008). The decision to classify a ‘mixed‐race’ patient as a ‘Black patient’ appears reminiscent of the so‐called ‘one drop rule’ in the US, which refers to the practice of using any evidence of African ancestry to categorise a person as ‘Black’ (Davis 1991). However, we should not overlook other elements of the topologies of race in this data, such as the (prompted) recognition of difficulties of categorising ‘mixed‐race’ patients and the desire to ‘monitor the effect of treatment’. These suggest that simplistic racial typologies can be folded together with more critical repertoires that question the validity of racial/ethnic categorisations, including for clinical practice.

In contrast to ‘mixed‐race’ patients, North African patients were not considered to be the ‘Black patients’ specified in the guidelines. Such judgments were sometimes based on visual cues. One clinician off‐handedly remarked that: ‘having been to Egypt on holiday, some of these people look more Asian’ (Respondent 7). Another elaborated: ‘I wouldn't treat them like a Black, no absolutely not. So probably a lot of it is visual actually […], It wouldn't occur to me to put somebody say Tunisian or Egyptian on you know the Afro Caribbean pathway’ (Respondent 9). In this extract classification is reported as almost obvious or self‐evident. Other clinicians, however, used more scientific terminology: ‘North Africans in terms of migration origin do not come into the same concept of African descent. We're talking about sub‐Saharan Africans’ (Respondent 8). As such, practices could be legitimated by reference to scientific evidence: ‘So Africa decision‐making ought to be based on whether the person has, are of, African decent, West African preferably because … although that Kenyan paper, and probably the South African data supports a general sub‐Saharan thing’ (Respondent 3).

As with the ‘mixed‐race’ example, respondents closed uncertainties by drawing together phenotypical distinctions and ideas about population prototypes that carry the authority of ‘common‐sense’; in this instance it was to decide that North Africans were not ‘Black patients’ of African descent. This can be interpreted as ‘common sense’ not only because it was based on visual cues, but also because the possibility of that allocation could be beyond everyday thinking (Posel 2001a, 2001b), as reflected in the respondent's comment that ‘it wouldn't occur to me’. However, there were also explanations of this allocation that invoked the authority of science. Some respondents justified the population‐level category ‘Sub‐Saharan Africans’ by reference to the epidemiology of (racialised) hypertension (e.g. Opie and Seedat 2005), and used terms like migration and descent from the ‘sciences of human origins’ (Schramm 2014: 3). Indeed, it is this articulation of racial/ethnic difference that underpinned the NICE (2006: 45) guideline's ‘hybrid’ phrase: ‘Black patients are those of African or Caribbean descent’. While ‘descent’ changed to ‘family origin’ in the guideline's 2011 update, the connotations of biological ancestry remain clear, and stand in contrast to the idea that census categories are manifestations of social identity.

## Discussion

Although the introduction of racialised categories in CPGs and clinical practice in this case was considered to be relatively unproblematic by most respondents in most instances, conceptual and socio‐political uncertainties about race/ethnicity were also apparent. In light of these findings, we discuss two interlinked questions. First, how is race/ethnicity articulated and enacted, including in the attitudes of our respondents? Second, how were inherent conceptual and socio‐political uncertainties ‘closed’ so that guidelines could be created and implemented?

One discussion about the potential social harms of using race/ethnicity in medicine centres on concerns about reifying racial/ethnic groups. Contemporary practices are contrasted with historical racial science for their focus on ancestry (not race), clines of genetic variation (not discrete racial categories) and ‘likelihoods’ of group differences (not essential characteristics) (Fujimura and Rajagopalan 2011). Our data on uncertainties relating to liminal cases, especially the expression of an over‐riding concern about monitoring treatment outcomes, lends some support to the idea that clinicians recognise conceptual complexity (Bonham *et al*. 2009). However, some of the ways in which race/ethnicity was enacted among our sample were decidedly ‘unreconstructed’: visual ascription (eyeballing), treating ‘mixed‐race’ as ‘Black’, and (sometimes tacit) ideas of a prototypical sub‐Saharan population type of true Africans. Other scholars have also noted these kinds of ad hoc judgments in assigning racial/ethnic identities to patients (Braun *et al*. 2007, Dyson *et al*. 2007, Hunt *et al*. 2013) or research subjects (Shim 2014). Most of our respondents thought that, in most cases, identifying race/ethnicity was an unproblematic act, because they either requested their patients to self‐identify to a standard classification, or they regarded their visual judgments as ‘common‐sense’ (Posel 2001a, 2001b). Yet, we saw the potential for ‘mis‐assignment’ (Braun *et al*. 2007, Dyson *et al*. 2007, Hunt *et al*. 2013) within the parameters of the guideline's classification when it came to the treatment of ‘mixed‐race’ patients. For our respondents, treating some ‘mixed‐race’ patients on the ‘Black patient’ pathway did not appear to raise concerns about mistreatment or error. However, even if the health risks are low in this clinical context (and this is not something we are in position to judge), this still raises issues about the consistency of practice when applying racialised guidelines, especially in relation to categories that may be ‘liminal’.

It has also been argued that contemporary racialised science is now, in part, informed by concerns about inequality (e.g. Fullwiley 2008, Montoya 2011). In our study, an ‘equality and diversity’ discourse was evident in the stakeholder consultation process. Furthermore, some interviewees (usually but not exclusively experts in epidemiology and public health) were overtly concerned by ethnic inequalities. While they advanced an ‘anti‐racist racialism’ (Bliss 2012), this centred more on health inequalities and less on the deconstruction of biological race, or the dangers of reification (Duster 2015). Concerns about reifying race/ethnicity as biological or natural rarely figured as a proximate or tangible risk relating to guideline development and never appeared in accounts of clinical decision‐making. We also found notable variations in how ideas about socio‐political sensitivities were articulated, including anxieties about saying ‘crude’ things and the dismissal or disparagement of a perceived imposition of ‘political correctness’. It is important to recognise the existence of both supportive and resistant attitudes toward ‘equality and diversity’ policies and the ambiguities in their articulation, as these could influence how racialised CPGs are perceived, understood or practiced.

Attending to the attitudes of professionals is important for understanding and shaping practice, and it also helps to demonstrate the novelty of contemporary contexts. However, a motivations‐based analysis risks pigeonholing ‘good’ and ‘bad’ scientists or clinicians based on their values/attitudes. Furthermore, we might ask how much (analytical) weight should be attached to equality‐orientated attitudes when the racialising practices of overtly equality‐oriented scientists or clinicians are substantially the same as those of others? Motivations, then, should be seen as only part of the analysis.

Our remaining analysis will focus on the social architectures that respondents drew upon to articulate and enact race/ethnicity. To do this, we pursue Pollock's (2012) claim that biomedical practitioners ‘close’ conceptual and socio‐political uncertainties about race/ethnicity in order to act in their professional capacities. This explanation is helpful, but it says relatively little about how this practice is facilitated, or how judgments are shaped. Here we return to the idea of the topologies of race, which works on ‘the presupposition that elements that are distant in time and space can become proximate and relevant in the here and now’ (M'charek *et al*. 2014b: 5). Uncovering the connections between disparate elements is important as it not only reduces the analytical emphasis on the motives of individuals, but also reveals how the CPGs for hypertension are enfolded with wider social processes of racialisation.

What elements are made ‘proximate’ in this case? Temporally, the guideline enrols 80 years of scientific thinking about racial/ethnic difference in hypertension, including theorising and investigating at the genetic, molecular, biological, somatic and epidemiological levels. Spatially, it aligns to research undertaken on racially/ethnically labelled populations from across the world, including the important ALLHAT trial that was designed to reflect a US socio‐political agenda that demanded ‘inclusiveness’. During guideline development, other socio‐political influences were woven into the CPG such as NICE's policy for using a national census tool; a classification which itself reflects locally situated ‘racial common‐sense’ (Posel 2001a, 2001b) and 30 years of contestation between state bureaucracy, minority ethnic groups and social scientists (Booth 1985, Sillitoe and White 1992). The stakeholder engagement introduced other context‐specific issues, including conceptual/operational and socio‐political concerns about implementation. The articulation of race/ethnicity in the final CPG folded together these various elements, and coined the hybrid designation: ‘Black patients of African or Caribbean descent’. When we asked how patients were (or should be) classified in order to implement the guidelines, responses reflected logics and techniques that invoked state bureaucracy (to request racial/ethnic self‐identification) and/or ‘common‐sense’ and scientific notions of phenotype and population prototypes (to observer‐assign race/ethnicity).

We suggest that our respondents ‘closed’ the inherent conceptual and socio‐political uncertainties of racialised prescribing by folding together these disparate elements to justifying a racialised approach. The elements of race that were articulated in our case study invoked authority from different sources: science, state or ‘common‐sense’. Yet it is the layering of these that created a formidable racialised logic. If one source of authority failed, another was on hand: if the science was vague, the state census was used; if the census classifications were not helping, ‘common‐sense’ was called upon. This layering of multiple sources of authority allowed emergent problems in the classification process to be ‘worked around’, and for racialised prescribing for hypertension to be normalised (cf. Bowker and Star 1999, Posel 2001a, 2001b).

We can further consider the place of CPGs in sustaining the topologies of race. Schramm (2015:53) directs our ‘attention to the nodes, holes and fissures through which the multiple connections between different sectors and their racialising effects become visible’. We argue that the racialised hypertension guideline is one such node as, following Latour's (1996) characterisation of nodes as having multiple dimensions and connections, it draws together elements from science, the state and ‘common‐sense’. Such nodes can be generative ‐ points of renewal and growth that reflect, entrench and create connections between ideas, practices, artefacts and social relations (Latour 1996). The guideline encourages racialisation as it is a material connection that authoritatively created a path for translating racialised research into racialised healthcare practice. Moreover, it generates fresh information flows and feedback loops (Hacking 1999) organised by racialised logic (clinical experiences, patient understandings, prescribing patterns, etc.). Enacting a racialised CPG reproduces, sustains and perhaps encourages particular ideas about racial/ethnic difference, and the populations defined therein.

What are the particular ideas and relations of racial/ethnic difference that are made, or made to ‘stick’ (M'charek 2014: 48) in our case? While we found some instances of nuanced thinking about race/ethnicity, it is largely essentialised notion of racial/ethnic difference that were reproduced. The creation and implementation of the CPGs involves a confirmation bias; selectively enacting ideas and practices that support racialised logics, while ‘working around’ or marginalising those that are contrary or critical. To this extent, using racial/ethnic categories in CPGs normalises racialised logics in medicine and healthcare practice, which may, in turn, feed into other essentialist discourses on racial/ethnic difference in science and society.

## Conclusion

We can use our study to propose some theoretical implications about racialisation in the context of health. We echo M'charek *et al*.*'s* (2014b) topological account of race, where the justifications for racialised thinking and action can be multiple, potentially intersecting, but not necessarily coherent. However, we emphasise that a triumvirate of powerful authority sources underpin the processes and logic of racialisation. Social actors can ‘close’ inherent conceptual and socio‐political uncertainties about race/ethnicity by overtly or tacitly invoking authority from science, the state and/or ‘common‐sense’. In the face of persistent uncertainty, ideas and practices that draw authority from different sources may be layered up to reinforce one another, or perhaps fused to create novel, hybridised articulations. Additionally, within this ‘live’ mesh of ideas and practices, certain artefacts act as nodes, or conduits, for perpetuating, expanding or transforming racialised logics. In formal contexts, like health care, such artefacts will be likely to claim the rationalist authority of science and/or the state (which is not to say that they are divested of ‘common‐sense’ ideas about racial/ethnic difference). We suggest that such artefacts are important to explanations of racialisation as they shed light on the persistence and the plasticity of ideas about racial/ethnic difference, and they perhaps offer a locus for interventions that seek to control or change such ideas.

Judging if our findings are ‘transferable’ to other health contexts would require further data, but we can tentatively suggest an implication of racialised CPGs. The goal of guidelines – to standardise practice – will be difficult to achieve because of the implicit conceptual and socio‐political uncertainties of race/ethnicity. Conceptual uncertainties give rise to operational difficulties, which could include problems like: naming categories and defining their boundaries; designing effective and efficacious classification schemes; ensuring a ‘fit’ between formalised schemes and everyday practice; and, providing training and/or guidance to ensure formal schemes are used consistently. Socio‐political uncertainties leave space for beliefs and values to enter into judgments. Whether practitioners think that it a ‘good thing’ or a ‘bad thing’ to design or use racialised CPGs could be influenced by personal or political differences, such as: perceptions and understandings of race/ethnicity; attitudes toward racial/ethnic inequalities; or, opinions of ‘equality and diversity’ or ‘anti‐racist’ agendas, policies or practices. It is clear that some professionals who produce or follow racialised CPGs will perceive them as relatively uncontroversial and intuitive. However, it seems inevitable that the implicit conceptual and socio‐political uncertainties of race/ethnicity will emerge, and when these are ‘closed’ to enable action (Pollock 2012) there will be differences in practice. The health risks of such variations or the inconsistent application of guidelines are likely to depend on clinical context.

## Note

1 Race and ethnicity are distinct but overlapping concepts. We adopt the term race/ethnicity to reflect the blurriness of the concepts and language used in our study. When citing the work of others we maintain their original terminology.
